# A Protein Complex Restrains a Homicidal Enzyme during Sperm Differentiation

**DOI:** 10.1371/journal.pbio.0050279

**Published:** 2007-09-18

**Authors:** Liza Gross

If one were to take a moral view of cell activities, the caspases would be condemned on two counts. They not only give the order to kill cells, they also carry it out. But in the real-life utilitarian world of the cell, all this killing is simply a means to an end. Programmed cell death, or apoptosis, plays an important role in embryonic development and in protecting the body from damaged or aged cells.

Caspases can also function without killing cells—for example, during inflammation, cellular differentiation, and morphogenesis—but even these nonlethal processes usually require the destruction of unwanted components. In the fruit fly Drosophila, for example, caspases facilitate the radical remodeling that liberates individual sperm from a cytoplasm-filled cyst containing 64 interconnected spermatids. During this process, called spermatid individualization, the cyst dispatches most of its cytoplasm and organelles into “waste bags” to yield the sleek DNA-delivery machines we call sperm.

What restrains the lethal machinations of caspases to allow selective degradation of subcellular components is not clear, though there is evidence that inhibitory proteins (called inhibitor of apoptosis proteins, or IAPs) may prevent caspase activation at inappropriate times. In a new study, Eli Arama et al. provide support for this scenario by showing that an enzyme complex activates caspases by degrading their inhibitors during Drosophila sperm differentiation. Although the complex has been linked to protein degradation before, its role in caspase regulation came as a surprise.

Almost every multicellular organism harbors caspase precursors that remain nearly dormant until aptly named activator proteins convert them into “initiator” complexes, which in turn activate “effector” caspases—the executioner enzymes. Once activated, effector caspases shear off protein fragments from a number of cellular targets, compromising their function and leading to apoptosis. IAPs can block caspase activity, and thus cell death, in insects and mammals. The Drosophila IAP (called Diap1) thwarts apoptosis by degrading an initiator caspase; Diap1 is inactive, however, in cells destined to die. *Diap1* encodes an enzyme (E3 ubiquitin ligase) that marks proteins for destruction by modifying them with small molecules called ubiquitin.

To determine which molecules rein in caspases’ lethal tendencies during sperm differentiation, the researchers analyzed a collection of over 1,000 lines of sterile mutants with defects in spermatid individualization. By isolating numerous strains with the same defect and crossing them in various combinations, researchers can identify “complementation groups” based on which strains fail to “complement,” or compensate for, the others’ deficit. The assumption is that each complementation group contains a different mutation responsible for the defect—thereby revealing the different genes required for the normal condition.

The researchers treated the testes of each of the mutant lines (each representing a different genetic mutation) with an antibody stain (CM1) that reveals the presence of cleaved (and thus active) caspase-3. Just 33 gene variants (or alleles), representing 22 complementation groups, lacked the CM1 stain and thus lacked active caspase-3. Since most of the sterile mutants retained the stain, despite having serious defects in spermatid individualization, the researchers concluded that caspase activation operates independently of other sperm differentiation pathways.

**Figure:**
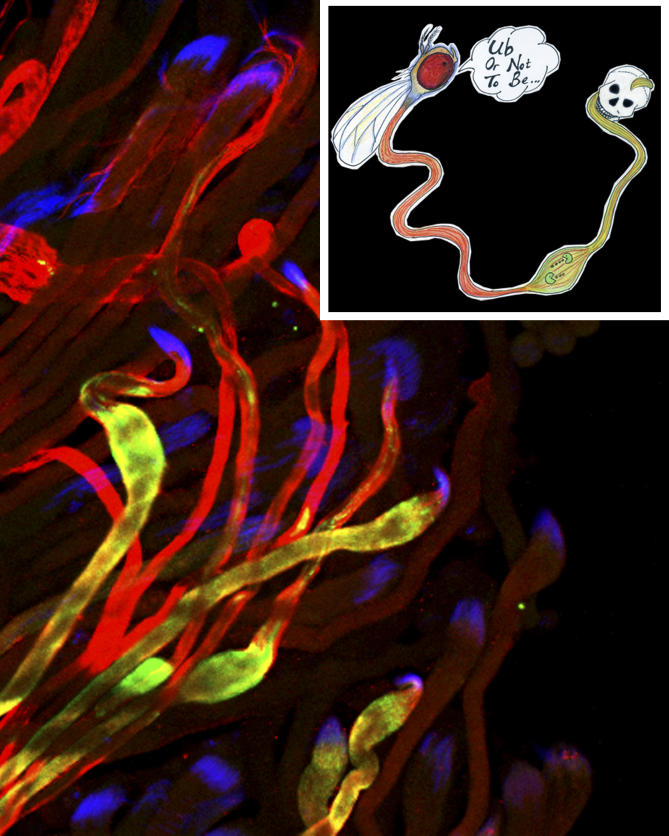
Drosophila spermatid “individualization” requires cell death proteins, including activated apoptotic effector caspases (stained green), and radically changes the cellular architecture. Individualizing spermatid tails are marked by the red staining of polyglycylated axonemal tubulin; the nuclear bundles are in blue. (Inset cartoon drawn by Anat Florentin and written by Yosef Kaplan, both in the Department of Molecular Genetics at The Weizmann Institute of Science)

To differentiate mutants that affect caspase activation from those that disrupt other elements of sperm differentiation, the researchers used a stain (AXO 49) that reveals when the sperm’s flagellar backbone (called axonemal tubulin) is modified, just at the onset of individualization. One group of defective strains, representing five different alleles, had the AXO 49 stain—indicating that the corresponding gene functions after the initial differentiation signals—and varying degrees of the CM1 stain. The researchers named the mutations medusa *(mds)* after the Greek mythic character symbolizing life and death. *mds1* had no sign of caspase activation, while the other four displayed various degrees of the stain.

The researchers mapped the mutations to a gene called *cullin-3* and demonstrated that the gene is required for caspase activity (by showing that testes lacking the *mds1* allele of *cullin-3* resembled those treated with a caspase inhibitor). Through “complementation tests” to better characterize the *mds* mutations, the researchers discovered that some *cullin-3* mutants, which were lethal, did not compensate for the sterility of the *mds* mutants—indicating that they represent a new class of mutations in *cullin-3*.

In contrast to the conventional wisdom that *cullins* are universally expressed, the researchers identified two slightly different products of *cullin-3* with tissue-specific expression—one is expressed only in the testes, and only in male germ cells, while the other is found only in body (soma) cells. The gene’s differential expression explains why some *cullin-3* mutations cause death while others cause sterility.

Through a series of gene deletion and protein interaction experiments, the researchers identified two proteins (Roc1b and Klhl10) that form a complex with Cullin-3 to promote caspase activation and spermatid individualization in the fruit fly. Altogether, their results suggest that the Cullin-3 complex adds ubiquitin tags to caspase inhibitors at the beginning of individualization, thereby dispatching the inhibitors and giving caspases free rein. What mechanisms keep the caspases from unleashing the cell death program remains an open question, though the researchers suspect that the localization of caspase inhibitor degradation may curtail apoptosis. In addition to identifying a new E3 ligase in the regulation of caspases, these results may also shed light on the origins of human infertility. Fruit flies and mammals share many aspects of spermatid individualization, and human fertility requires the Cullin-3 enzyme complex.

